# Habitat Analysis of Breast Cancer-Enhanced MRI Reflects BRCA1 Mutation Determined by Immunohistochemistry

**DOI:** 10.1155/2022/9623173

**Published:** 2022-03-30

**Authors:** Tianming Du, Haidong Zhao

**Affiliations:** Department of Breast Surgery, Second Affiliated Hospital of Dalian Medical University, 467, Zhongshan Road, Shahekou District, Dalian, Liaoning 116023, China

## Abstract

**Objective:**

To use habitat analysis (also termed habitat imaging) for classifying untreated breast cancer-enhanced magnetic resonance imaging (MRI) in women. Moreover, we intended to obtain clustering parameters to predict the BReast CAncer gene 1 (BRCA1) gene mutation and to determine the use of MRI as a noninvasive examination tool.

**Methods:**

We obtained enhanced MRI data of patients with breast cancer before treatment and selected some sequences as the source of habitat imaging. We used the *k*-means clustering to classify these images. According to the formed subregions, we calculated several parameters to evaluate the clustering. We used immunohistochemistry to detect BRCA1 mutations. Moreover, we separately determined the ability of these parameters through independent modeling or multiple parameter joint modeling to predict these mutations.

**Results:**

Of all extracted values, separation (SP) demonstrated the best prediction performance for a single parameter (area under the receiver operating characteristic curve (AUC), 0.647; 95% confidence interval (CI), 0.557–0.731). Simultaneously, models based on the Calinski-Harabasz Index and sum of square error performed better in the training (AUC, 0.903; 95% CI, 0.831–0.96) and verification (AUC, 0.845; 95% CI, 0.723–0.942) sets for multiparameter joint modeling.

**Conclusion:**

Based on the enhanced MRI of breast tumors and the subregions generated according to the habitat imaging theory, the parameters extracted to describe the clustering effect could reflect the BRCA1 status. Differences between clusters, including the general differences of cluster centers and clusters and the similarity of samples within clusters, were the embodiment of this mutation. We propose an algorithm to predict the BRCA1 mutation of a patient according to the enhanced MRI of the breast tumor.

## 1. Introduction

In 2020, the number of new breast cancer cases worldwide was 2.26 million, and breast cancer has replaced lung cancer as the most common cancer type. Moreover, breast cancer is the most common cancer leading to death in women, both in developing and developed countries [1]. Like all cancers, it is considered a heterogeneous type, and the biological behavior, treatment response, and clinical outcomes are closely related to the molecular typing of breast cancer determined by the estrogen receptor (ER), progesterone receptor (PR), human epidermal growth factor receptor 2 (HER2), and KI-67. Breast cancer is categorized by the following molecular classification: Luminal A, Luminal B, HER2 overexpression, and triple negative. The risk factors of breast cancer with different molecular classifications demonstrate considerable differences. For example, triple-negative breast cancer often displays a younger age of onset, compared with the most common Luminal A breast cancer. Of several risk factors, genetic susceptibility is the most important factor and is often associated with race [2]. It can become explicit with the environmental background and heredity. According to statistics, 20% to 40% of hereditary breast cancers can be attributed to harmful mutations in BReast CAncer gene 1 (BRCA1) and BReast CAncer gene 2 (BRCA2) [3]. In addition, 90% of BRCA1-associated breast cancers reveal negative ER expression [4]. Meanwhile, PR expression in BRCA1-associated tumors is lower than that in sporadic tumors [5], thus making breast tumors in patients with BRCA1 mutations more likely to be classified as triple-negative breast cancer, characterized by the loss of ER and PR expression and HER2 overexpression. Patients with this molecular subtype are considered to have poor prognosis. Triple-negative breast cancer has a shorter survival than other subtypes, with a 40% mortality within 5 years of diagnosis. Furthermore, it is highly invasive biologically, with distant metastasis occurring in approximately 46% of patients with triple-negative breast cancer. Following metastasis, the median survival time is only 13.3 months, and the postoperative recurrence rate is 25% even after surgery. In addition to the above-mentioned clinical manifestations [6, 7], BRCA1 mutations are pathologically associated with basal-like phenotypes in triple-negative breast cancer [8, 9].

The BRCA gene is involved in double-stranded DNA damage repair through homologous recombination; therefore, women with this mutation lack the tumor suppressor protein to repair damaged DNA [10]. BRCA proteins are essential for chromosomal stability, and their primary function is to protect the genome from damage. A recent study further suggests that BRCA transcription regulates numerous genes involved in DNA repair, cell cycle, and apoptosis. These functions are mediated by several cellular proteins that interact with BRCA, besides an association with phosphorylation events. Women with BRCA mutations have a higher risk of breast cancer at a younger age than those without [11]. Furthermore, this mutation is associated with an increased risk of ovarian cancer [12].

Patients with BRCA-associated breast cancer have a higher risk of ipsilateral and contralateral breast cancer recurrence with worse prognosis, compared with sporadic breast cancer. Despite numerous distinct clinical particularities of BRCA-associated breast cancer, current treatment recommendations are substantially similar to those for sporadic breast cancer, including surgery, radiation, and chemotherapy. However, BRCA1/2-associated breast cancer is accompanied by a defect of homologous recombination repair function, which may be more sensitive to DNA-damaging drugs, such as platinum drugs or poly-ADP ribose polymerase inhibitors. With increased tumor specificity, chemotherapy regimens may be used more for patients with BRCA mutations [13].

Immunohistochemistry (IHC) is an inexpensive and effective technique easily available to the majority of pathologists. It has been considerably used in several tumors. Researchers have made substantial progress in detecting BRCA1 mutations in digestive system cancer to predict its prognosis in response to chemotherapy [14]. Some studies have also suggested the possibility of using IHC in breast tumors [15].

Enhanced magnetic resonance imaging (MRI) is a sensitive breast imaging detection method [16]. Compared with mammary gland molybdenum target image and ultrasound, enhanced MRI has higher resolution and can observe the tissue perfusion status. Clinicians can use different functional MRI sequences to measure spatial differences in the cell density, tissue structure, perfusion, and metabolism. For people aged 25 years to 29 years with BRCA mutations, the NCCN guidelines recommend annual breast MRI screening or annual mammography [17]. Currently, there are some MRI studies on BRCA mutations in patients with breast cancer. The primary research method is based on first-order features and features extracted from gray-level cooccurrence matrix, gray-level run-length matrix, and gray-level size zone matrix. Those studies combined the features with the clinical information of patients to establish model [18, 19]. However, these methods predominantly explore the BRCA mutation imaging on the entire tumor but do not consider the tumor heterogeneity reflected by imaging.

Habitat analysis, also termed habitat imaging, is an imaging technology aimed at capturing subtle differences among tumors. It uses several algorithms to segment the tumor and its surrounding environment, aiming to obtain subregions which reflect the heterogeneity of the tumor [20]. In other words, this image-processing technique focuses on similar subregions within the tumor formed by the pressure of survival. In this study, we used the cluster parameters extracted from the habitat imaging of patients with enhanced MRI to predict BRCA1.

## 2. Materials and Methods

### 2.1. Study Participants

The radiology database of the Second Affiliated Hospital of Dalian Medical University was reviewed. We identified 187 patients who underwent MRI from March 2018 to June 2021. Fifty-one and 11 patients were excluded owing to an incomplete image sequence and the absence of distinct BRCA1 results. Therefore, a total of 125 patients were included in this study. The inclusion criteria were as follows: (i) no breast diseases before imaging examination and (ii) did not receive treatment for breast cancer or may artificially change breast imaging. The patients underwent MRI examinations before the surgery and were diagnosed as grades 3, 4, and 5 according to the Breast Imaging Reporting and Data System. Surgery or biopsy was performed within 1 week to confirm primary breast cancer diagnosis and BRCA1 mutation. We excluded specific breast malignancies, such as inflammatory breast cancer, Paget's disease, and breast cancer because of metastasis. Moreover, men and pregnant women were excluded. There was no specific information about patients in the study, so the study did not involve ethical issues.

### 2.2. Enhanced MRI

We used the American GE1.5 T Signa HDxt MRI scanner, and the receiving coil was a special one for the surface of the breast. In the prone position, the bilateral breast naturally hung in the concave hole of the coil. The scan sequence and parameters were as follows: diffusion-weighted imaging, *b* − value = 800 s/mm, repetition time (TR) = 5,600 ms, echo time (TE) = 74.4 ms, matrix 130 × 128, field of view (FOV) = 33 cm × 33 cm, and layer thickness 5 mm. All patients underwent dynamic contrast-enhanced MRI following DWI sequence scanning. Gadolinium diamine was used as the contrast agent. The injection volume was 0.2 mmol/kg, and the flow rate was 2 ml/s to 3 ml/s. Following injection, 20 ml normal saline was used to flush the tube. We performed continuous nonstop scanning. T1-weighted image plain scanning was initially performed. Following gadolinium injection, we continuously scanned nine phases, each phase of 47 s. A total of 10 phases were scanned. The scanning time was 7 min 6 s, and the turning angle was 15°. Other scanning parameters were as follows: TR = 5.1 ms, TE = 2.5 ms to 12 ms, matrix 320 × 384, FOV = 30.2 cm × 30.2 cm, and layer thickness 5 mm. Two radiologists with >10 years of experience in breast imaging diagnosis independently interpreted the MRI results. After discussing the images, they reached a diagnostic consensus. All data have been transferred to the GE workstation (Advantage Windows 4.5, General Electric, Madison, WI, USA).

### 2.3. Habitat Generation

**Figure 1:**
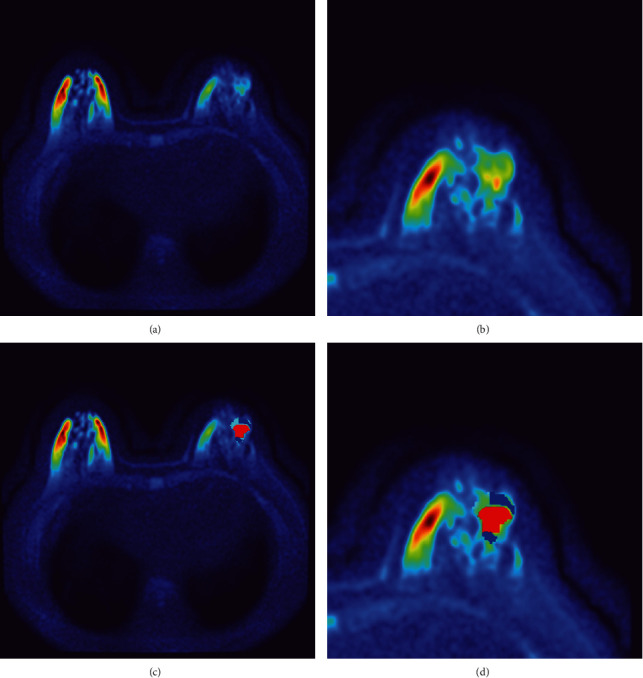
An example of habitat imaging. (a) A pseudocolor map of a 45-year-old patient with breast cancer using diffusion-weighted imaging. Following image diagnosis, the breast tumor (considered malignancy) has been confirmed by postoperative pathology. (b) The picture only depicts the segment containing the tumor. (c) The graph depicts three different subregions (three colors) following habitat analysis. (d) The picture only depicts the segment containing the tumor.

The Pydicom Library of Python was used to read the spatial location of the image. First, we used the nearest neighbor interpolation to process the images into pictures with equal pixel spacing, followed by image registration according to the spatial location. The region of interest (ROI) was manually obtained on a high-signal DWI, and there was no necrosis or cystic component under ideal circumstances. In the case of no satisfactory image (usually because of low resolution), the ROIs were drawn by referencing T1 images. According to the 3-D MRI images, we developed a segmentation algorithm with wide adaptability. First, we applied the Markov random field segmentation method to voxel data combined with the interaction between voxels, considering 27 individual elements around each voxel (3 × 3 × 3). Images of the DWI sequence, T1 sequence, OutPhase T2 sequence, InPhase T2 sequence, WATER T2 sequence, and FAT T2 sequence were standardized according to the gray value in ROIs to form new images to be clustered [21]. For each voxel in the ROI, the gray value in each image was used as a six-dimensional coordinate and the proportion of each weight was similar. The *K* clustering *k*-means algorithm was used for image segmentation (Figure 1) [22] in the following steps: (1) we selected *K* points as the initial cluster center; (2) for each sample in the dataset, we calculated its Euclidean distance to the *K* cluster centers and divided it into the class corresponding to the center with the smallest distance; (3) for each category, we recalculated the cluster center (i.e., the centroid of all samples belonging to the category); and (4) we repeated steps 2 and 3 until reaching a termination condition, such as iteration times and minimum error change. The final clustering results were evaluated using the maximum expectation. For the processed 3-D tumor ROI, we divided each tumor region into three spatially limited subregions with different characteristics using the aforementioned algorithm [23].

### 2.4. Immunohistochemistry

Pathological specimens were obtained from the enrolled patients. No data were excluded owing to missing values or ambiguity. All specimens were fixed with 4% neutral formaldehyde, embedded in paraffin, and continuously sectioned at a distance of 4 *μ*m. Following IHC staining, the specimens were observed and photographed under a microscope. We used the immunohistochemical SP method to detect the expression of susceptible genes. Specific steps were performed according to the standard instructions, and professional pathologists interpreted the films. We determined the comprehensive staining intensity and the percentage of positive cells. The final results were divided into the following categories: <5% visible staining: -; 5% to 25% visible staining: +; 26% to 50% visible staining: ++; and >50% visible staining: +++, where - was defined negative and +, ++, and +++ were defined positive.

### 2.5. Clustering Features

The clustering index was extracted from the clustering method to the following five items:

1. Inertia, within cluster sum of square error

For each cluster, we calculated the distance between the samples in the cluster and the cluster center and added the values to describe the similarity between each cluster.

(2) Calinski-Harabasz Index

The Calinski-Harabasz Index is defined as the ratio of discrete between groups to that within groups. The larger the value of the index, the smaller the distance within the cluster. The greater the distance between clusters, the better the clustering effect. The distance within the cluster is represented by that between the sample point within the cluster and the center point of the cluster.

(3) Silhouette coefficient

The silhouette coefficient was used to describe the cohesion and separation of clusters and defined the average distance between the point and other points within the cluster. The final silhouette coefficient is the average of silhouette coefficient of each sample. Its value ranges from -1 to 1, and the value is approximately 1 for a considerably greater separation degree between clusters than the cohesion. The closer the value to 1, the better the clustering effect.

(4) Separation

The separation value was obtained by calculating the arithmetic average of the sum of distances between each two cluster center points. In contrast to compactness, the indicator only considered the distance between different clusters. The larger the number, the better the clustering effect.

(5) Davies-Bouldin Index

The greater the distance between clusters and the smaller the distance within the cluster, the smaller the value is. The smaller the value of the Davies-Bouldin Index, the better the clustering performance.

## 3. Results

### 3.1. Clinical Features

The patient's clinical information is as follows (Table 1).

### 3.2. Features and Prediction Model

**Figure 2:**
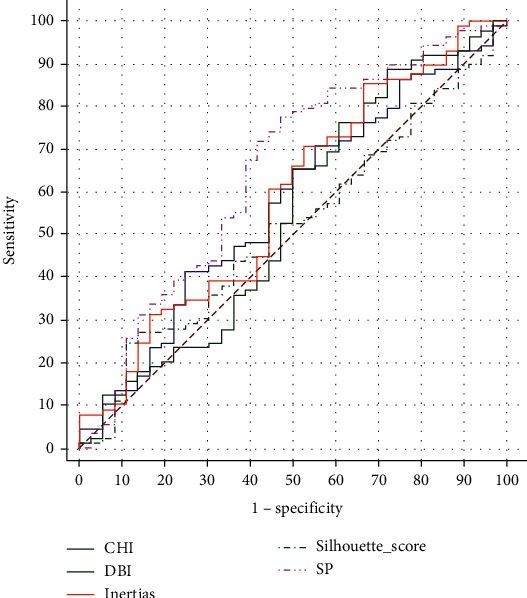
Receiver operating characteristic for clustering features.

SP demonstrated excellent predictive power for the clustering features (area under the receiver operating characteristic curve (AUC), 0.647; 95% confidence interval (CI), 0.557–0.731) (Figure 2).

**Figure 3:**
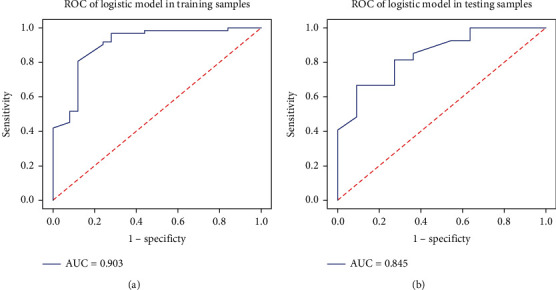
Receiver operating characteristic of the prediction model: (a) the performance of training set; (b) the performance of the validation set.

Then, we take the AdaBoost algorithm for modeling, the weak classifier is Gaussian process, and we select two characteristics as the modeling parameters, respectively, Calinski-Harabasz Index and Inertia. The prediction model also displayed good diagnostic efficiency (training group: AUC, 0.903; 95% CI, 0.831–0.96; accuracy, 0.897; specificity, 0.72; and sensitivity, 0.968; test group: AUC, 0.845; 95% CI, 0.723–0.942; accuracy, 0.789; specificity, 0.636; and sensitivity, 0.852) (Figure 3).

**Figure 4:**
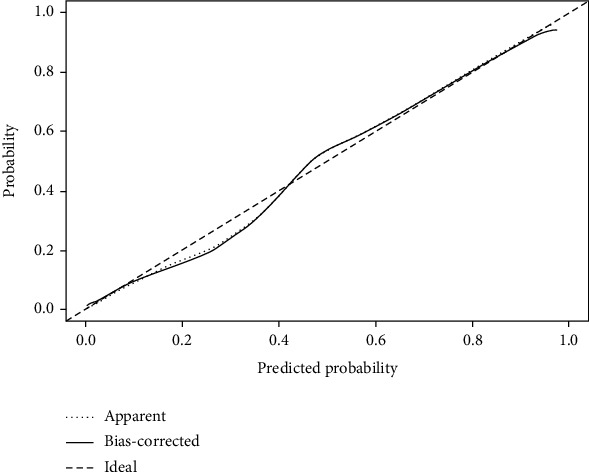
Calibration curve of the prediction model.

We plotted the calibration curve for the prediction model (Figure 4). Our prediction model displayed stability and considerable prediction ability.

## 4. Discussion

In this study, we classified the breast cancer image into three subregions by using the gray distribution of multiple sequences of images of patients. After this, we use various parameters to identify BRCA1 mutations, which can assist pathological diagnosis. For example, despite a large staining area that can be recognized by naked eyes, the degree of staining is shallow and difficult to count by equipment. In this case, it is difficult for pathologists to diagnose BRCA1 by only pathological slices, and this method can provide an auxiliary diagnostic basis.

BRCA1 mutations affect the incidence, progression, diagnosis, treatment, and prognosis of breast cancer throughout the course of the disease [24]. Simultaneously, BRCA mutation-associated breast cancers are more likely to develop lymph node metastases. In addition to macroscopic changes in clinical manifestations, such as morbidity and prognosis in terms of tumor heterogeneity, tumors with BRCA1 mutation usually display invasive ductal carcinoma pathologically and have a higher incidence of myeloid carcinoma and atypical myeloid carcinoma [25]. Despite their role in the auxiliary diagnosis of breast cancer, it is relatively difficult to make a preliminary judgment only by imaging. In ultrasound examination, BRCA1 and BRCA2-mutated breast cancer demonstrates an irregular shape and blurred boundary hypoechoic masses. However, unlike BRCA2, 29.5% of BRCA1 mutations display posterior echo enhancement, usually in benign breast lesions, such as fibroadenomas or cysts. The aforementioned feature may lead to breast cancer with BRCA1 mutation being misdiagnosed as a benign tumor to some extent [26]. Invasive breast cancers with BRCA1 mutations do not reveal more calcification on breast cancer-specific mammography. In terms of the medical information obtained, MRI is a significantly better imaging method than ultrasound and molybdenum target. However, it partially addresses the problem of BRCA1 displaying benign tumor characteristics in ultrasound. This is because a radiologist can obtain additional information in MRI, including internal enhancement and hemodynamics. However, in this study, we identified some invasive breast cancers with smooth margins and other manifestations similar to benign masses. Nonetheless, BRCA1 breast cancers have obvious edge enhancement, which enables excluding fibroadenoma diagnosis. This marginal enhancement results from peripheral vascular proliferation and central necrosis. In summary, it is difficult to make qualitative diagnosis by only relying on the experience of the imaging physicians and basic influencing parameters. This warrants the development of a noninvasive, qualitative imaging diagnostic model. Radiology research at present is more inclined to generate a ROI first and then extract texture information from it, which considers the tumor as a whole. Finally, the researchers explore the relationship between texture and patients' clinical information by machine learning and other methods. Some previous experiments were based on the theory of habitat imaging, but only on animal models [18]. Some study based on the theory of habitat imaging, but after researchers segmented images, they chose to extract the texture information of each subregion, and then established a neural network model by those texture information. This method emphasizes more the differences between subregions than the connections. Undoubtedly, characterizing the entire tumor as an indivisible whole is not the most sensitive method for assessing the intratumor heterogeneity. Habitat analysis lends its name to the definition of an ecological habitat surrounding a population of species. This emerging approach explicitly divides tumors containing similar characteristics into subregions. Such differences are not only from macroscopic features, such as necrosis caused by uninhibited growth, but also from significant genetic heterogeneity between and within tumors [27]. This is usually attributed to random mutations that drive cloning and evolution [28]. Nonetheless, tumor cells can promote independent growth owing to local angiogenesis, which will lead to chaos in the distribution of blood vessels in the tumor and cause tumor blood flow within random and cyclical changes. Eventually, it will cause the uneven distribution of oxygen, glucose, hydrogen ions, and serum growth factors. Differentially acting tumor cells lead to the specific evolution of tumor population to adapt to this environment [29, 30]. Based on this theory, the imaging results of tumors can predict the associated genetic factors as well as the characteristics of tumor populations generated under specific circumstances [31]. This in turn allows habitat imaging to strongly explain the different outcomes in patients with similar molecular subtype, following an identical therapeutic strategy [17]. Moreover, habitat imaging plays an important role in explaining the causes of drug resistance during chemotherapy in a particular patient. For example, changes in the composition of tumor subareas often attribute to the failure of patients in achieving pathological complete response during neoadjuvant chemotherapy. Meanwhile, the development of drug resistance in a specific subregion leads to poor therapeutic response in therapy. This phenomenon has expanded the use of habitat imaging from screening people at a high risk of cancer to assessing the treatment and patient outcomes throughout the disease. Currently, habitat imaging has made good progress in neural tumors [32].

Different from traditional imaging omics studies, we classified the tumors into subregions and analyzed their relationship to explain spatial heterogeneity. We consider both the differences between each cluster and the cluster shape. In this study, we extracted five parameters of a term used to describe the heterogeneity of cancer. These parameters were used for the simultaneous differentiation between each tumor subregion, thus considering the similarity between each cluster sample. In other words, our parameters can describe each cluster more effectively. Moreover, it described the degree of heterogeneity within the tumor from various angles in detail, thus providing a description of the tumor. We focused not only on the absolute characteristics of the tumor but also on the relative relationship between the subregions in case of individual differences. Some habitat imaging studies use images of tumors as samples separately or simply select the largest cross-section of the image as a sample. The aforementioned method can achieve certain advantages in sample representativeness. Nonetheless, tumors do not have a fixed shape and growth direction like some tissues or organs. In other words, tumors do not have a homogeneous and stable physiological and anatomical structure. Partial cardiac tumor necrosis and uneven texture distribution (i.e., tumor heterogeneity) are the major pathological changes. Moreover, necrosis can predict the effect of tumor therapy [33]. Thus, cross-sectional images do not represent the structural characteristics of the entire tumor. The selection of the largest cross-section will result in imbalance of the segmentation. However, it will introduce an error for the experimental method that principally considers the relative relationship of the subregion. In fact, all five of the parameters we showed can be affected by this. Therefore, the 3-D image of the whole tumor needs to be used as the segmentation object. In summary, the proportion of each subregion of the entire tumor, cluster shape, and cluster center affected the modeling, thereby indicating the need for considering the tumor as a whole.

Despite considering several aspects, there were several shortcomings in this study. First, the use of a specific imaging protocol for enhanced MRI, not common in clinical practice, was a major limitation. Despite attempting to standardize the imaging and selecting fewer additional multicenter data and the validation set, technical factors, such as the field strength, repetition time, echo time, and flip angle, may have affected the results. This warrants greater and larger multicenter studies to confirm and validate our findings. Another limitation was in terms of image registration. One benefit of the imaging protocol was that the image sequence could be easily registered, for each sequence of patient images was generated at the same time. There is still a lot of work to be done to roll out the technology. Manual recalibration following the rough registration of the algorithm is a feasible method to ensure the consistency of the voxel represented by similar coordinates. In addition, we excluded a significant number of patients, which resulted in the inclusion of fewer patients, particularly owing to the absence of prospective cases. In the image processing, we only adopted a filter to remove the image noise, which was justified from the results. By contrast, the oversegmented technology [34] has been used by researchers to initially divide the tumor into smaller blocks and subsequent clusters, which may be more helpful in describing the real growth of the tumor. However, despite not adopting the oversegmented technology, we considered the distribution of local tissue abnormalities during image processing. Thus, we used the mean value of peripheral voxels within the range of 3∗3∗3 to replace each voxel, which may help us offset the impact of noise. A reasonable theory is that clusters formed by our approach were more representative, provided greater space continuity. The oversegmented technology can be more detailed and generate an accurate depiction of the tumor tissue heterogeneity distribution. However, the application of the oversegmented technology may result in two situations. Fewer number of clusters will deteriorate the continuity of the segmented subregions. By contrast, increasing the number of clusters will lead to the poor representation of some subregions, thus posing challenges to the complexity of subsequent modeling. Because of the above-mentioned technical difficulties, obtaining satisfactory training results necessitates the use of more powerful modeling methods, such as neural network. By contrast, the model should be trained with a larger amount of data to avoid overfitting problems. The selection of the neural network and the inclusion of excessive data should be avoided in this experiment. In addition, the weights of all sequences in the clustering were equal and fixed during the final use of image clustering. We did not consider the redundancy of clinical information displayed by different sequences. The 3-D image segmentation method resulted in limited amount of data. Therefore, we adopted the efficient clustering method, which eventually led to overfitting. Hence, we used the image data to the maximum possible to provide various parameters for clustering. This may have led to a high proportion of information describing some aspect of the clustering effect, i.e., the relationship between subregions, thereby affecting the accuracy of clustering. In the final modeling stage, the adoption of greater parameters could not improve the final effect of the model. It also shows that the redundant information interfered with the model.

## 5. Conclusions

In summary, a model is established based on clustering parameters extracted from subregions formed by habitat imaging. The model can predict BRCA1 mutations detected by immunohistochemistry satisfactorily. And it provided a reference for screening the high-risk population, surgery, drug therapy, and prognosis.

## Figures and Tables

**Table 1:** Clinical characteristics of patients with breast cancer.

Characteristics	Training group (*n* = 100)	Test group (*n* = 25)
Age (years)	55.2	56.1
ER		
Positive	80	19
Negative	20	6
PR		
Positive	67	18
Negative	23	7
HER2		
Positive	31	5
Negative	69	20
Stage I	69	20
Stage II	23	4
Stage III	5	1
Stage IV	3	0
Lymphonodus		
Positive	30	8
Negative	70	17
BRCA1		
Positive	69	20
Negative	31	5

ER: estrogen receptor; PR: progesterone receptor; HERK2: human epidermal growth factor receptor 2; BRCA1: BReast CAncer gene 1.

## Data Availability

The MRI and clinic Information data used to support the findings of this study are available from the corresponding author upon request.
